# A hidden cause of infertility in hypothyroid patients

**DOI:** 10.1002/ccr3.2654

**Published:** 2020-01-14

**Authors:** Magdy Mohamed Allam, Hanaa Tarek El‐Zawawy, Sherine Samir Barakat, Soha Magdy Ahmed, Rasha Noureldin M. Saleh

**Affiliations:** ^1^ Internal Medicine Department, Endocrinology Unit Alexandria University Student Hospital (AUSH) Alexandria Egypt; ^2^ Internal Medicine Department Faculty of Medicine Alexandria University Alexandria Egypt; ^3^ Internal Medicine Department Haematology Unit Alexandria University Student Hospital (AUSH) Alexandria Egypt; ^4^ Clinical Pathology Department Faculty of Medicine Alexandria University Alexandria Egypt; ^5^ Norwich Medical School University of East Anglia Norwich UK

**Keywords:** 5‐methyl tetrahydrofolate, Hashimoto's thyroiditis, hypothyroidism, infertility, methylene tetrahydrofolate reductase, miscarriage, pregnancy loss

## Abstract

Methylene tetrahydrofolate reductase (MTHFR) gene mutations could be the cause of infertility in hypothyroid patients. Hence, it is worthy to screen for MTHFR gene mutations in infertile hypothyroid females and their partners if infertility persists after optimizing thyroid function.

## Graphical abstract

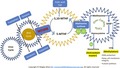

## 1 INTRODUCTION

Thyroid gland dysfunction carries a detrimental effect on endometrium, ovulatory function, and fertility (from implantation to delivery).^1^ The incidence of miscarriage, mainly occurs in the first trimester, was found to be higher (6%‐15%) in patients with hypothyroidism compared to normal population (2.2%) with a higher risk of miscarriage as a result of the increase in maternal TSH concentrations. Apart from thyroid function, TPOAb were found to be associated with an increased miscarriage rate up to 43.9%.^2^

Many mechanisms have been studied to explain the cause of infertility and subfertility in hypothyroid patients including associated hyperprolactinemia, gonadotropins ovarian axis effects,^3^ direct ovulatory dysfunction,^4^ and endometrial dysfunction (endometrium‐blastocyst interaction).^5^

However, the underlying pathological mechanisms remain unclear. Hypotheses suggest that autoimmune thyroiditis may be a part of an immune‐mediated disorder or an autoimmune polyglandular syndrome, which includes autoimmune vasculitis of the placental microvasculature.^6^ In addition, antithyroid antibodies can directly cause hypothyroidism in newborns via crossing the placental barrier.^7^

Recent guidelines recommend that thyroid status and TPOAb level to be taken into consideration separately regarding levothyroxine (LT4) treatment in infertile women especially those who are undergoing assisted reproductive techniques (ART).^8^ However, a considerable number of studies which showed that appropriate LT4 treatment (targeting TSH level <2.5 mIU/L) has not been proven to be effective in management of infertility in many hypothyroid patients.^9^, ^10^

This study reports two cases of hypothyroidism with a long history of infertility in both primary (first case) and secondary due to repeated pregnancy loss (second case) who were suffering from infertility after optimization of the euthyroid state (TSH below 2.5 mIU/ml). After methylene tetrahydrofolate reductase (MTHFR) gene mutation was detected, the patients were able to conceive till full‐term when 5‐methyl tetrahydrofolate (5‐MTHF) was supplemented.

## 2 CASE PRESENTATION

### 2.1 Case one

A 26‐year woman was referred from the gynecology clinic to our endocrinology clinic. Her main complaint was inability to conceive for 1.5 years. She had no relevant medical history. She had regular menses. Regarding her family history, her father was on treatment for hyperthyroidism.

On examination, her body mass index (BMI): 24.3 kg/m^2^, and blood pressure (BP): 90/60 mm Hg. Her thyroid gland was not palpable.

Laboratory investigations showed: TSH = 5.66 mIU/mL (0.39‐4.16 mIU/L), FT4 = 1 ng/dL (0.8‐2 ng/dL), FT3 = 2.4 pg/mL (1.4‐4.2 pg/mL), and TPOAb > 600 IU/mL (0‐40 IU/mL). Ultrasound neck revealed normal thyroid gland volume with heterogeneous hypoechoic pattern.

Based on the aforementioned results, the patient was diagnosed with Hashimoto's thyroiditis with subclinical hypothyroidism. The patient was maintained on LT4 (75 mcg/d). After 3 months, her test results showed: TSH = 1.32 μIU/mL (adjusted according to ATA guidelines to be <2.5 mIU/L), FT4 = 1.29 ng/dL, FT3 = 2.7 pg/mL.

The patient became pregnant after three failed trials of induction of ovulation using clomiphene citrate despite a good ovarian response. During pregnancy, 5 mg/d folic acid and LT4 100 mcg/d were prescribed. However, she had a miscarriage at 15th week of gestation.

Infertility workup showed that her partner had normal sperm parameters according to WHO criteria, hysterosalpingography and transvaginal ultrasonography revealed no abnormalities.

Hormonal profile showed: FSH = 5.8 mIU/mL (normal; 3.5‐12.5 mIU/mL), LH = 6.6 mIU/mL (normal; 2.6‐12.6 mIU/mL), PRL = 8 ng/mL (normal; 2‐18 ng/mL), and AMH = 4 ng/mL (good fertility; 1.68‐4.4 ng/mL) which were optimal for fertility. Immune markers including antinuclear antibody (ANA), antidouble stranded DNA antibody (anti‐dsDNA), lupus anticoagulant (LA), and anticardiolipin antibody were all negative. In addition, antithrombin III, protein C, and protein S were negative.

After 6 months of follow‐up, keeping TSH below 2.5 μIU/mL (optimal for pregnancy), she got pregnant again. Low‐dose aspirin, enoxaparin, LT4, and folic acid 5 mg were prescribed. Miscarriage occurred at 14th week of gestation, despite the last TSH was 0.7 μIU/mL, FT4 = 0.5 ng/dL, FT3 = 2.8 pg/mL, and TPOAb = 297 IU/mL.

After 9 months of follow‐up, keeping TSH below 2.5 μIU/mL, TPOAb progressively increased to its previous level (>600 IU/mL). Infertility workup revealed that the patient was heterozygous for C677T in methylene tetrahydrofolate reductase (MTHFR) gene with a normal serum homocysteine (9.8 μmoles/L) level. Consequently, a supplement containing 5‐methyl tetrahydrofolate (5‐MTHF) 800 µG daily, which supported the one‐carbon cycle (Zn, B3, B6, B12) with cobalamin, low‐dose aspirin and LT4 100 mcg/d were prescribed.

Two months later, she got pregnant. During the pregnancy, she was maintained on low‐dose aspirin and LT4 and 5‐MTHF with cobalamin. She gave birth to a healthy female baby. Thereafter, TSH = 1.23 μIU/mL, FT4 = 1.15 ng/dL, FT3 = 2.53 pg/mL, and TPOAb = 482 IU/mL at 6 months after delivery. At 24 months after delivery, TPOAb titer declined further.

### 2.2 Case two

A 24‐year old woman, who had a six‐year‐old child, was complaining of failure to conceive for 2 years. She was diagnosed with hypothyroid due to Hashimoto's thyroiditis 3 years ago. Induction of ovulation using clomiphene citrate failed five times, despite good ovarian response. Her medical history only included chronic allergic rhinitis. She was maintained on LT4 100 μg. She had been suffering from menorrhagia for 6 years. Her family history was irrelevant.

On examination, her BMI: 32.3 kg/m^2^ and BP: 90/60 mm Hg. Her thyroid gland was not palpable. TSH = 3.6 μIU/mL, FT4 = 1.1 ng/dL, FT3 = 2.7 pg/mL, and TPOAb = 1950 IU/mL. Ultrasound neck revealed normal thyroid gland volume with heterogeneous hypoechoic pattern. Upon that she was diagnosed as Hashimoto`s thyroiditis.

LT4 was titrated till reaching a dose of 125 mcg/d. After 3 months, TSH = 0.92 μIU/mL, FT4 = 1.69 ng/dL, and FT3 = 2.7 pg/mL. In spite of maintaining a TSH level below 2.5, the patient still did not conceive. Infertility workup for the patient and her husband showed that CASA analysis was normal according to WHO parameters, hysterosalpingography and transvaginal ultrasonography revealed no abnormalities. Hormonal profile revealed that FSH = 4.9 mIU/mL, LH = 4.6 mIU/mL, PRL = 10.3 ng/mL, and AMH = 3.7 ng/mL which were optimal for fertility. ANA, anti‐dsDNA, LA, antithrombin III, protein C, and protein S were all negative.

The couple had experienced two failed invitro fertilization (IVF) cycles, with nine metaphase II oocytes injected, six oocytes fertilized, and a total of two embryos transferred. During all of the IVF attempts, she was prescribed DHEA 25 mg three times a day and a high (5 mg/d) dose of folic acid.

Interestingly, the patient was homozygous for MTHFR A1298C and her husband was heterozygous for MTHFR C677T mutation. A supplement containing 5‐MTHF (Zn, B3, B6, B12), low‐dose aspirin, and LT4 100 mcg were prescribed. MTHF was prescribed for the husband as well.

She got pregnant after 2 months of keeping TSH below 2.5 μIU/mL and TPOAb level decreased to 357 IU/mL. During pregnancy, she was maintained on low‐dose aspirin, LT4 150 mcg, enoxaparin 40 mg, and MTHF 500 mg with cobalamin 1000 mg.

Unfortunately, the patient stopped the treatment on her own after 12 weeks, after which she delivered a baby with multiple congenital anomalies who eventually died 3 weeks later.

Six months later, the patient sought medical advice again at our clinic and after explaining the importance of her compliance on MTHF plus LT4, enoxaparin 40 mg, and aspirin, she got pregnant after 4 months.

During her pregnancy, she was maintained on the same previous medications with the same doses. At the 13th week of gestation, enoxaparin dose was raised to 60 mg due to poor placental perfusion indicated by high resistance index (RI) of retro chorionic blood flow (0.33). Eventually, she gave birth to a healthy female baby.

## 3 DISCUSSION

### 3.1 MTHFR mutation and female infertility

**Figure 1:**
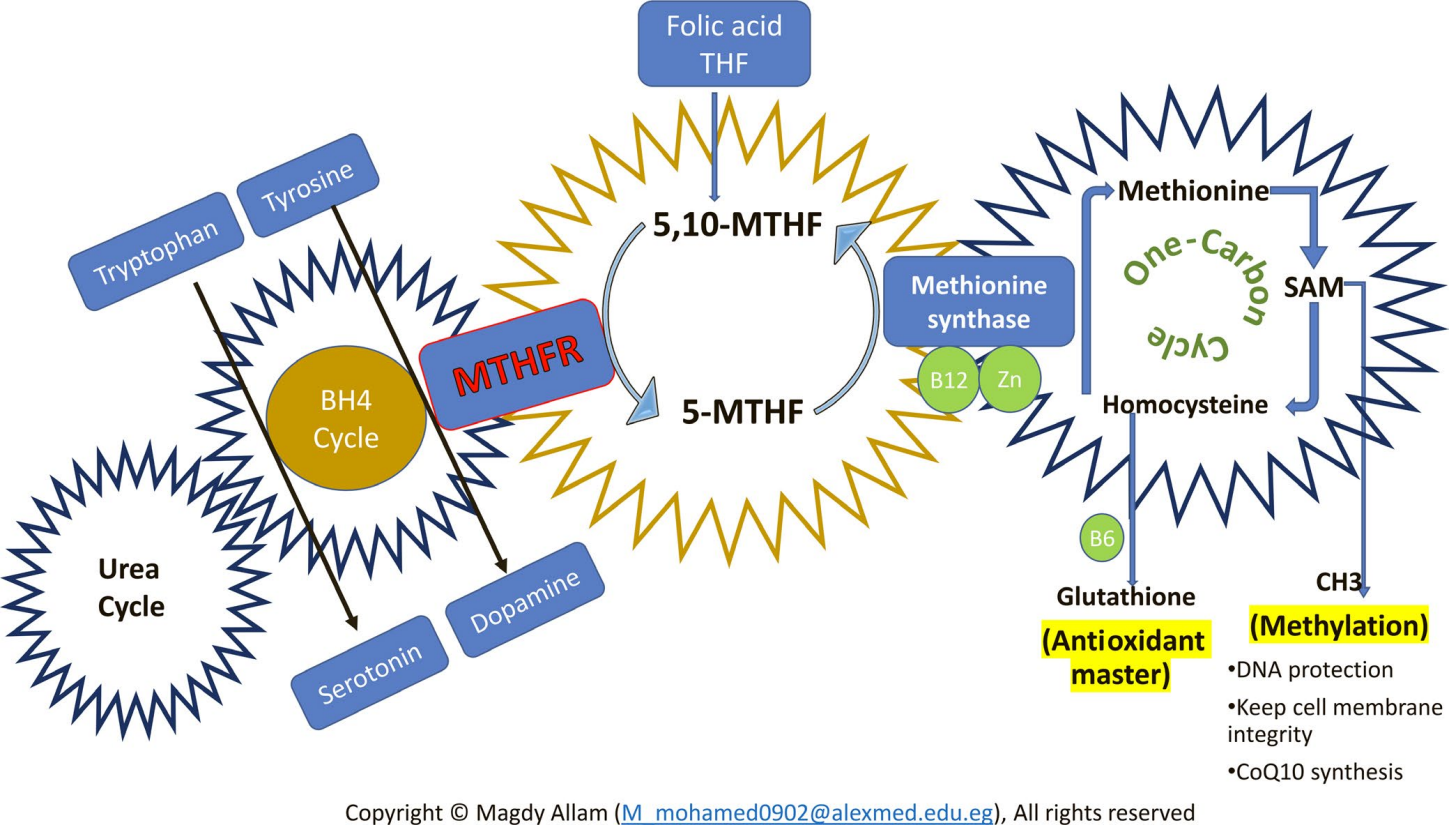
Schematic figure showing the process by which folate/folic acid is used for methylation & antioxidation. MTHFR, methylenetetrahydrofolate reductase; SAH, S‐adenosylhomocysteine; SAM, S‐adenosylmethionine; THF, tetrahydrofolate

For decades, folate is known to be essential for reproductive health. Not only due to its importance in oogenesis and spermatogenesis but also in prevention of perinatal complications, mainly neural tube defects.^11^ MTHFR is a crucial enzyme involved in the folate cycle and one‐carbon cycle. It catalyzes the irreversible conversion of 5, 10 MTHF to 5‐MTHF, which serves as a methyl donor in the remethylation of homocysteine to methionine. The methylation process aids in processing amino acids for neurotransmitters, detoxification of compounds like homocysteine (Hcy), antioxidation (by glutathione/ hypotaurine) and DNA repair (Figure 1).

A missense mutation in exon 4 of the MTHFR gene, a cytosine‐to‐thymine substitution at position 677 (C677T) that converts an alanine codon to a valine codon, causes thermolability of MTHFR.^12^ A polymorphism in exon 7 of MTHFR results from an adenine‐to‐cytosine substitution at position 1298 (A1298C).^13^

MTHFR polymorphisms were observed. African Americans appear to be protected from MTHFR deficiency. Hispanics and Caucasians may be at elevated risk due to increased frequencies of C677T and A1298C, respectively.^14^ Based on a global reference for human genetic variation, approximately 25% of the global population are carriers of MTHFR C677T, Hispanics being the population with the highest frequency (47%), followed by Europeans (36%), East Asians (30%), South Asians (12%), and Africans (9%). Approximately 13.5% of Europeans are homozygous for the variant allele. Also, MTHFR A1298C is present in about 25% of the global population and occurs with higher frequency in South East Asians (42%) and Europeans (31%), followed by Hispanics and Africans is 15%. Approximately 11% of Europeans are homozygous for the 1298A > C variant allele.^15^

MTHFR mutations, especially for C677T and A1298C, decrease the enzyme capacity to form 5 MTHF to 70% and 40%, respectively.^16^, ^17^ Moreover, these mutations are associated with recurrent miscarriages, which occurs particularly with large doses of folic acid (4‐5 mg/d).^18^ The mechanism of MTHFR‐related poor pregnancy outcomes may related to hyperhomocysteinemia, premature ovarian failure,^19^ thrombophilia,^20^ trophoblastic affection,^21^ preimplantation chromosomal anomalies,^22^ and male spermatogenesis affection.^23^

### 3.2 MTHFR gene mutation in hypothyroid pregnant female: relation to folic acid

Pregnancy is known to be a state of low folic acid level. As during pregnancy, folate is required for rapid cell proliferation and tissue growth of the uterus and placenta, fetal growth, and expansion of the maternal blood volume.^24^

Moreover, hypothyroidism has always been associated with low folic acid level and a state of hyperhomocysteinemea (more than 100%).^25^ Experimental studies showed that MTHFR activity was low in hypothyroidism and high in hyperthyroidism.^26^

In addition to fortifying food with folate, the US Preventive Services Task Force recommends that all women be prescribed multivitamins containing folic acid at least 1 month prior to conception to prevent neural tube defects,^27^ and increase a woman's chances of becoming pregnant.^28^ To our knowledge, there are no guidelines regarding folic acid supplementation during the perinatal period in patients with thyroid dysfunction.

Nevertheless, the relationship between the MTHFR mutation and infertility is controversial whether in natural pregnancy or ART.^29^ Even though, it has not been recommended to screen for MTHFR gene mutations for outcome of pregnancy in ART.^30^

Based on our cases, it is suggested that the combined interaction of hypothyroidism, pregnancy, and MTHFR mutations may have a synergistic effect on folic acid metabolism which will eventually affect pregnancy outcomes and fertility in both, male and females.

Finally, the average direct costs of both patients for delaying diagnosis of MTHFR gene mutation including of doctor visits, cost of medications, cost of failed ICSI procedure, hospital admissions, and cost of laboratory tests without inclusion of psychological burden are 90 000 LE. On contrary, cost of MTHFR test and treatment for a year is 2100 LE. So, it is cost‐effective to screen infertile hypothyroid patients for MTHFR gene mutations.

## 4 CONCLUSION

To our knowledge, this is the first time alarm directed to MTHFR mutations as a suggested cause of infertility and miscarriage in hypothyroid patients and their partners. This neglected cause could be treated with MTHF supplementation for MTHFR carriers couples.

## CONFLICT OF INTEREST

The author state that there are no conflicts of interest.

## AUTHOR CONTRIBUTION

MMA: participated in patient management, data collection and writing the draft, and critically reviewed the manuscript. HTE: participated in data collection, in writing the draft. SSB: contributed to the interpretation of the cases and critically reviewed the manuscript. SMA: critically reviewed the manuscript. RNMS: contributed to the interpretation of the cases and critically reviewed the manuscript.

## INFORMED CONSENT

Written informed consent was obtained from the patients for publication of this case report.
